# Minocycline fails to modulate cerebrospinal fluid HIV infection or immune activation in chronic untreated HIV-1 infection: results of a pilot study

**DOI:** 10.1186/1742-6405-8-17

**Published:** 2011-05-12

**Authors:** Emily L Ho, Serena S Spudich, Evelyn Lee, Dietmar Fuchs, Elizabeth Sinclair, Richard W Price

**Affiliations:** 1 Department of Neurology1 University of California San Francisco, San Francisco, CA, USA; 2 Division of Biological Chemistry, Biocentre, Innsbruck Medical University, Innsbruck, Austria; 3 Division of Experimental Medicine, Department of Medicine, University of California San Francisco, San Francisco, CA, USA; 4 Department of Neurology, University of Washington, Seattle, WA, USA; 5 Department of Neurology, Yale University, New Haven, CT, USA

## Abstract

**Background:**

Minocycline is a tetracycline antibiotic that has been shown to attenuate central nervous system (CNS) lentivirus infection, immune activation, and brain injury in model systems. To initiate assessment of minocycline as an adjuvant therapy in human CNS HIV infection, we conducted an open-labelled pilot study of its effects on cerebrospinal fluid (CSF) and blood biomarkers of infection and immune responses in 7 viremic subjects not taking antiretroviral therapy.

**Results:**

There were no discernable effects of minocycline on CSF or blood HIV-1 RNA, or biomarkers of immune activation and inflammation including: CSF and blood neopterin, CSF CCL2, CSF white blood cell count, and expression of cell-surface activation markers on CSF and blood T lymphocytes and monocytes.

**Conclusions:**

This pilot study of biological responses to minocycline suggests little potential for its use as adjunctive antiviral or immunomodulating therapy in chronic untreated HIV infection.

## Background

Human immunodeficiency virus type one (HIV) infection of the central nervous system (CNS) is a nearly ubiquitous facet of systemic infection that begins early after exposure [1-6]. This CNS infection is accompanied by local immune responses that are reflected in elevations of CSF biomarkers of immune activation and inflammation [7-11]. Though clinically inapparent in most patients, CNS HIV infection evolves in some to a more 'invasive' HIV encephalitis (HIVE) that manifests with the cognitive and motor dysfunction characteristic of the AIDS dementia complex (ADC) [12], now commonly referred to as HIV-associated dementia (HAD) [13]. While the pathogenesis of brain injury related to HIVE is not precisely understood, it likely involves 'indirect' pathways of injury in which host inflammatory mediators serve as important neuropathogenic signals and toxins and, hence, in a broad sense can be considered immunopathological [14,15]. Chronic subclinical CNS infection may also be accompanied by more indolent brain injury that manifests later as cognitive impairment [13,16,17] and possibly continues despite antiretroviral treatment [18]. Although the pathogenesis of this type of chronic injury is less well understood than that of HIVE, continued immune activation may be an important factor [8,19,20].

These indirect mechanisms of injury have led to a search for *adjuvant *modes of treatment to mitigate brain injury by attenuating immunopathology or interfering with downstream neurotoxic pathways. While a number of adjunctive therapies have been advocated or tested [21], none of these has yet proved effective or entered clinical practice. Recently, the antibiotic, minocycline, has been proposed as a candidate therapy in this broad class. Minocycline has been shown to reduce lentivirus infection and immune responses in model systems [22-27] and also to exert neuroprotective effects in diverse models of neurodegeneration [28-35]. This has led to the suggestion that it might be useful in human HIV infection, either as an adjunct to [25] or low-cost replacement for antiretroviral treatment, with particular relevance to attenuation of CNS infection and disease. To begin to test this in the human disease setting, we initiated a pilot study to evaluate minocycline in chronic human HIV infection in the absence of antiretroviral therapy, using CSF and blood biomarkers as principal indices of drug effects, with CSF infection thus serving as a 'model' of and window into CNS infection and immunoactivation [36,37]. For this open-labelled pilot study we hypothesized that minocycline would reduce CSF HIV-1 RNA concentrations, both absolutely and in relation to blood HIV-1 RNA, and diminish evidence of CSF and blood immune activation, including CSF and blood concentrations of neopterin [11,38], CSF concentrations of CCL2 (monocyte chemotactic protein-1, MCP-1) [39,40] and T cell and monocyte expression of cell-surface activation markers [10].

## Results

Of 17 subjects screened over a period of 3 years (2006-2009), 6 were excluded because of low CSF HIV-1 RNA (N = 3) or unsuccessful lumbar punctures (N = 3). Three other subjects withdrew from the study without starting minocycline treatment. One subject enrolled in the study but stopped after 4 days due to a reaction to minocycline (nausea and vomiting) that resolved after stopping the drug. The remaining 7 subjects entered the study and were prescribed minocycline. Their baseline characteristics are shown in Table 1. Six of these completed the study without adverse events. One subject discontinued minocycline after week 4 of the study due to elevations in serum transaminases, but continued study participation through the washout period and the last visit at week 14; the transaminases subsequently returned to normal. For repeated measures ANOVA analysis, this subject's 4-week results were carried forward and included in the 8-week data. The six remaining subjects tolerated the treatment without clinical or laboratory evidence of toxicity.

**Table 1:** Baseline subject characteristics

	***Median***	***Range***
	
**Age **(years)	49.9	32.0 - 55.2
		
**Gender **(M:F)	6:1	
		
**Time since HIV diagnosis **(years)	17.0	1.7 - 20.3
		
**HIV-1 RNA **(log_10 _copies/mL)		
Plasma	4.49	4.26 - 5.56
CSF	3.87	3.11 - 4.47
Plasma:CSF difference	1.06	0.12 - 1.58
		
**Blood T cells **(cells/μL)		
CD4+	453	267 - 806
CD8+	1,009	575 - 2185
		
**CSF WBCs **(cells/μL)	9	2 - 18
		
**Neopterin **(nmol/L)		
CSF	13.1	5.9 - 41.2
Plasma	13.4	9.2 - 53.5
		
**CSF CCL2 **(pg/mL)	479.2	397.9 - 1322.2
		
**T Cell Activation **(percent CD38+/HLA-DR+)		
CSF CD4+	14.7	3.4 - 60.2
Blood CD4+	13.3	7.6 - 24.7
CSF CD8+	83.4	41.8 - 97.6
Blood CD8+	58.5	34.5 - 78.0
		
**Monocyte Activation **(percent CD16+)		
CSF monocytes	93.6	80.1 - 100
Blood monocytes	10.8	4.7 - 17.0
		
**CSF:blood albumin ratio**	5.05	3.91 - 12.26
		
**QNPZ-4**	-0.32	-3.44 - 0.54

**Figure 1:**
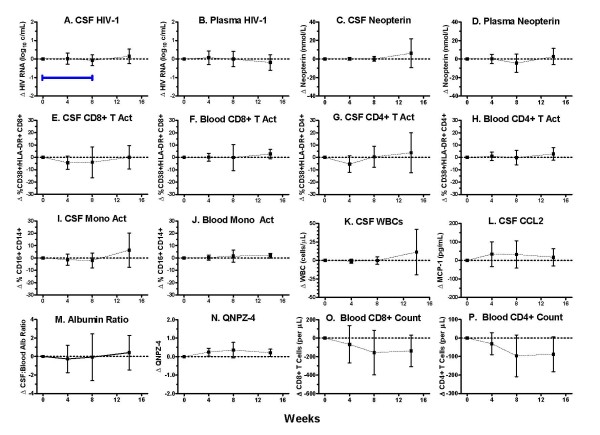
**Changes in outcome variables in the CSF and blood with minocycline treatment**. The horizontal bar in panel A indicates the period of minocycline treatment. Panels show the mean changes from baseline and 95% confidence intervals for CSF (A) and plasma (B) HIV-1 RNA concentrations; CSF (C) and plasma (D) neopterin concentrations; percent of CSF (E) and blood (F) CD8+ T cell activation, as assessed by co-expression of CD38 and HLA-DR on CD3+CD8+ lymphocytes; percent of CSF (G) and blood (H) CD4+ T cell activation, as assessed by co-expression of CD38 and HLA-DR on CD3+CD4+ lymphocytes; percent of CSF monocyte activation (I) as assessed by CD16 expression on CD14+CD4loCD3lo cells; percent of blood monocyte activation (J) as assessed by CD16 expression on CD14+CD4loCD3- cells; CSF WBC counts (K); CSF CCL2 concentration (L); QNPZ-4 performance score (N); and blood CD8+ (O) and CD4+ (P) T cell counts. Analysis of individual changes from baseline by Kruskal-Wallis and Dunn's post hoc testing from baseline to 8 weeks or 14 weeks and by repeated measures from baseline to 8 or 14 weeks with Dunnet's post hoc testing of each interval found no significant changes for any of the 12 variables shown except for changes in the blood CD4+ T cell counts (P), which was statistically significant for weeks 0 - 8 (P = 0.035) and weeks 0 - 14 (P = 0.013). Abbreviation: Act = activation.

Figure 1 shows the changes from baseline in the primary and secondary outcome measures. There were no significant changes in the virological measures. Both the CSF (A) and plasma (B) HIV-1 RNA remained stable, as did the CSF:plasma HIV-1 RNA ratio (not shown). Likewise, neither the CSF (C) nor plasma (D) neopterin changed. Similarly, none of the CSF or blood T cell (E - H) or monocyte (I and J) activation levels changed. There was no reduction in the CSF WBC count (K), which is composed principally of blood-derived T cells [10,41,42]. CSF CCL2 (L), CSF:blood albumin ratio (M), and the brief measure of neurological performance, the QNPZ-4 score (N), also did not change significantly. Curiously, there was a reduction of absolute CD8+ (O) and CD4+ (P) T cell numbers in the blood, although only the latter was statistically significant by repeated measures analysis.

## Discussion

This pilot study was undertaken to explore the use of minocycline as an adjuvant treatment for chronic HIV infection, particularly for attenuating the CNS components of immunoactivation and infection. It aimed to provide a preliminary view of the biological effects of minocycline on CNS HIV immune reactions and infection, and to obtain effect-size estimates for power calculations prior to planning a larger controlled trial. Our underlying mechanistic hypotheses centered on the proposed capacity of minocycline to attenuate CNS immune and systemic perturbations and their effects on CNS infection as revealed by changes in CSF and blood biomarkers. We hypothesized that attenuating these immunological effects would be reflected in reductions in CSF (and perhaps plasma) neopterin and CSF CCL2 concentrations, and in the expression of surface activation markers on T cells and monocytes. Additionally, we hypothesized that minocycline might also indirectly reduce CNS infection through its effects on various immune system-related mechanisms that contribute to the magnitude of CNS (and CSF) infection, including: CD4+ T cell traffic that brings both infected cells and uninfected targets into the CNS, and CD4+ T cell and macrophage activation that enhance viral replication in these cell types. Unfortunately, in this study, none of these effects were seen. Similarly, there were no changes in the other secondary endpoints, including CSF WBC counts, CSF:blood albumin ratios or the brief neurological performance battery, the QNPZ-4.

Minocycline, a licensed tetracycline antibiotic, has been reported to have a number of properties that make it an attractive adjuvant therapy candidate. In various model systems, it has been shown to have anti-inflammatory effects [43,44], including modulation of T cell activation and attenuation of macrophage and microglial activation [27,34,45,46]. It also has neuroprotective properties *in vitro *and in *in vivo *animal models [47-52]. These and other properties have led to trials of minocycline in several conditions, including rheumatoid arthritis [53], and neurodegenerative and neuroinflammatory diseases [33,52,54,55]. Minocycline also has been shown to inhibit HIV replication in microglia *in vitro *[22]. Importantly, in an SIV model of accelerated CNS infection, minocycline-treated SIV-infected macaques were noted to have less severe encephalitis, reduced expression of CNS inflammatory markers, reduced axonal degeneration and lower levels of CNS virus replication [23]. Recent *in vitro *studies on human peripheral blood CD4+ T cells demonstrate that minocycline has anti-viral effects in CD4+ T cells and reduces cellular CD4+ T cell activation [27]. Since all of these properties made it an intriguing candidate for adjuvant use in CNS HIV infection, our study results thus beg the issue of why we did not see similar effects in the studied patients.

While it is possible that the CSF measurements were insensitive to salutary effects on the brain parenchyma, including the important perivascular environment, this does not seem likely. CSF neopterin is a marker of CNS macrophage activation (presumably including both brain and meningeal populations) that increases with disease severity and is especially elevated in HIVE/HAD [11,38]. This pteridine biomarker responds well to antiretroviral therapy [11], although it does not always return to normal levels [8,19,56]. Its blood concentration is also a prognostic marker of disease progression [57]. Both CSF and blood levels were unaffected by minocycline in our study, suggesting that there was little effect on CNS or systemic macrophage activation. Similarly, CSF CCL2, a biomarker of macrophage chemotaxis that is also characteristically elevated in HAD/HIVE [58], showed no changes. This is especially disappointing since CSF CCL2 has been used as a biomarker in SIV encephalitis, and was shown to be reduced by minocycline treatment in the SIV model [23,40]. Increased levels of CD4+ T cell, CD8+ T cell and monocyte activation observed in the CSF compared to the blood is characteristic of HIV infection [10,42,59] and is likely an important component of both systemic [60-62] and CNS disease pathogenesis [10,20]. These measures also were stable through the course of minocyline treatment.

CSF HIV-1 RNA levels reflect more than one cellular source, with the relative contributions differing depending on the stage of systemic and CNS infection and disease evolution [63-66]. Short-lived cells, presumably CD4+ T cells, contribute a CSF viral population that is genetically similar to the blood population [63]. This component has been termed *transitory *infection [5,37] and is presumably sustained by infected and susceptible CD4+ T cells trafficking into the meninges and brain. In early HIV infection, this type of infection predominates and may even be the only type detected [67]. A second viral population turns over more slowly [66]. This population is likely derived from macrophages, and is genetically distinct from the blood population. This component, termed *autonomous *or compartmentalized infection, is characteristically detected as a minor contributor to CSF HIV levels in neuroasymptomatic chronic infection, but predominates in more advanced infection, particularly HIV encephalitis (HIVE) [64].

Minocycline might attenuate both types of infection by its effects on T cell and monocyte-macrophage activation. In the case of transitory infection, T cell activation is critical to support HIV replication and also promotes T cell traffic that carries infected and uninfected target CD4+ T cells into the meninges and perivascular spaces. Hence, if minocycline alters these T cell properties it might reduce this type of CSF infection. Similarly, activation is likely important for macrophages in sustaining infection and also, perhaps, in their entry into the CNS, including into the perivascular spaces, meninges and parenchyma. Minocycline might, therefore, reduce this type of autonomous infection. However, we detected no evidence of reduced CSF infection, although in the subjects studied with relatively preserved blood CD4+ T cell counts, the major CSF viral population likely originated from transitory type infection, although this was not directly examined in these subjects.

Our methods of examining the hypothesized actions of minocycline should have been adequate to detect a substantial immunological or virological effect of minocycline. Possible reasons as to why there were no discernable effects similar to those in the SIV-infected pigtailed macaques may have included species differences. Perhaps more likely were differences in the disease targets. The SIV model differs from our subjects in the relatively short disease duration and the presence of frank lentivirus encephalitis [23]. Our study patients had a chronic 'stable' infection for a number of years and thus, perhaps, presented a level of immune activation and viral replication that the drug effect was too weak to modify. In addition, the absence of encephalitis meant that there might have been little CNS disease to target. Our study, of course, did not address these possibilities.

The study also did not assess the more direct neuroprotective properties of minocycline. With one exception, our subjects were largely neuroasymptomatic, and we performed only brief quantitative neurological performance testing (QNPZ-4) on four measures. The small improvement noted in this measure, which was not statistically significant, might have related to practice effect. However, if the observed improvement was indeed real, then a study with 20-25 subjects in each of two treatment arms (minocycline and placebo) would be needed for an 80% power to detect the difference found here at 8 weeks. An AIDS Clinical Trials Group study is studying whether minocycline might improve performance in cognitively impaired HIV-infected subjects (http://clinicaltrials.gov/ct2/show/NCT00361257), and these issues should be addressed by that study.

The observed decline in blood CD4+ and CD8+ T cell counts was unexpected and unexplained. Curiously, it did not impact the CSF WBC count. This mild T-cell lymphopenia needs to be verified in a larger study, and if so, subject to further investigation.

Overall, this pilot study was subject to several inherent design limitations, including its small size, relatively short duration, and absence of an untreated control group for comparison, raising concern for Type II error. Thus, we cannot fully dismiss the possibility that the study was underpowered to detect a mild effect of the drug or that CSF HIV and CNS immune activation might decline further with longer exposure. However, given the minimal changes noted in the major outcomes, it would take a large study to test the effectiveness of minocycline on these measures in this type of patient population. For example, if the small reduction (-0.070 log10 copies/mL) in CSF HIV-1 RNA at 8 weeks was indeed a 'real' finding, then it would require more than 100 subjects in each of the two arms (minocycline and placebo) to have an 80% power to detect this difference between the groups, a difference with likely little clinical meaning. In the case of CSF neopterin, there was no statistically significant reduction, but if the slight increase at 8 weeks (0.033 nmol/L) was inverted and actually a reduction, it would take 500 subject in each group to detect this difference. Thus, the effects of minocycline on infection and immune activation appeared too weak to justify a study of the requisite size, particularly when viewed in comparison to the potent effects of combination antiretroviral on these variables [6].

## Conclusions

In conclusion, this small pilot study suggests that any effects of minocycline on CNS HIV infection and immune activation were not sufficient to impact chronic HIV in the absence of antiretroviral treatment. Therefore, there seems little justification or indeed ethical basis for treating chronic HIV infection with minocycline *instead of *combination antiretroviral drugs. However, given the reported *in vitro *and SIV effects of this tetracycline [23], there still may be reason for further study, for example in well-treated patients in which the level of immunoactivation is partially attenuated or in patients with cognitive impairment in which its neuroprotective properties may yet prove useful in concert with combination antiretroviral treatment.

## Methods

This study was approved by the University of California San Francisco Committee on Human Research and conducted according to the principles expressed in the Declaration of Helsinki. Informed written consent was obtained from all subjects. The study was registered with ClinicalTrials.gov (number: NCT01064752).

### Study design

This was an open-labelled, uncontrolled, pilot study examining the effects of 100 mg of minocycline taken orally twice daily for 8 weeks. Subject entry criteria included: ≥18 years of age; chronic HIV infection with plasma and CSF HIV-1 RNA concentrations >1,000 copies/mL; not taking antiretroviral therapy (either naïve to therapy or >6 weeks off treatment with no plans to start during the period of study); predicted medication adherence; blood CD4+ T cell counts >100 cells/μl; no previous adverse reaction to tetracyclines; no tetracycline treatment for the past 6 months; no contraindications to lumbar puncture (LP); no active opportunistic infection or neurological disease confounding evaluations; ADC stage <1 [68]; no concomitant medications altering the metabolism or risk of minocycline; hemoglobin >10 g/dL and liver transaminases <2.5 times upper limit of normal; and not taking any other immunomodulating drugs. After consent, subjects underwent a screening evaluation that included lumbar puncture (LP) and CSF characterization, concurrent blood sampling, and standardized neurological assessments as previously described [6,10,69]. For those meeting entry criteria, this also served as the baseline visit, and they starting minocycline 100 mg twice daily orally for the next 8 weeks. At four and eight weeks, and after a 6-week washout period off minocycline, subjects underwent repeated evaluation similar to the baseline, including LP and CSF analysis [6,10,69]. Treatment adherence was assessed at each on-study visit by direct questioning and pill count.

The primary outcome measures were the change from baseline during treatment in CSF HIV-1 RNA and CSF neopterin concentrations as indices of CNS infection and immunoactivation [38]. Change from baseline was calculated at weeks four and eight after initiation of minocycline treatment and after a 6-week wash-out period. Additional secondary measured outcomes included changes in: CSF white blood cell (WBC) count; blood CD4+ and CD8+ counts; ratio of CSF to blood albumin as a measure of blood-brain barrier permeability [70,71]; CSF CCL2 as a measure of monocyte-macrophage chemotaxis [58]; CSF and blood CD4+ and CD8+ T cell and monocyte activation as measured by multiparameter flow cytometry [10]. Four quantitative tests (timed gait, grooved pegboard, finger tapping and digit symbol) were used to obtain a simple quantitative neurological performance aggregate score (QNPZ-4) [72].

### CSF and blood assays

HIV-1 RNA was measured in cell-free CSF and plasma by the Roche Amplicor HIV-1 Monitor assay (versions 1.0 and 1.5, Roche Diagnostic Systems, Inc., Branchburg, N.J). Neopterin concentrations in cell-free CSF and plasma were measured in batch by ELISA according to the manufacturer's instructions (BRAHMS Aktiengesellschaft, Hennigsdorf, Germany). Blood CD4+ and CD8+ T cell counts were performed in the San Francisco General Hospital (SFGH) Clinical Laboratories using standard flow cytometric methods. CCL2 was measured in cell-free CSF by ELISA (R&D Systems, Minneapolis, MN). Other measurements performed in the SFGH Clinical Laboratories using routine clinical methods included CSF and blood albumin (used to compute the CSF:blood albumin ratio [70,71]), CSF WBC counts and differential, CSF total protein and blood metabolic profile.

CSF and blood CD4+ and CD8+ T cell activation were assessed by the percent of these cells in fresh specimens co-expressing surface CD38 and HLA-DR by multiparameter flow cytometry as previously described [10]. Blood monocytes were defined as CD14+CD4loCD3- cells from the mononuclear gate. CSF monocytes had low level staining for CD3 and were defined as CD14+CD4loCD3lo cells. Monocyte activation was defined by the percent of these cells expressing CD16 [10]. Flow cytometry data was compensated and analysed with FlowJo (Tree Star, Ashland, OR).

### Statistics

Changes from baseline to follow-up test intervals were analysed by Kruskal-Wallis test with Dunn's post hoc comparison of individual intervals and additionally from baseline through week 8 using repeated measures ANOVA with Dunnet's post hoc comparison. All P values were two-sided with values <0.05 considered significant. Statistical analyses used Prism 5 (GraphPad Software Inc, San Diego, CA) while power calculations used StatMate 2.00 (GraphPad Software Inc).

## Competing interests

Dr. Price has received funding from Merck to support an investigator-initiated research study and an honorarium from Abbott for a conference presentation. The other authors have no competing interests.

## Authors' contributions

ELH examined study participants, performed lumbar punctures, and assisted with the analysis of the data and preparation of the manuscript. SSS examined study participants, performed lumbar punctures, and assisted in design of the study and reviewed the manuscript. EL served as the patient study coordinator, aided in the design of the study, performed the quantitative neurological performance testing and managed the data. DF performed assays of CSF and plasma neopterin. ES designed the flow cytometry assays, directed the SFGH Clinical Immunology Laboratory that performed the flow cytometry assays and CSF CCL2 ELISA assays, and analysed and interpreted flow cytometry data. RWP designed and oversaw the study, examined study participants, performed lumbar punctures, analysed and interpreted the data, and participated in preparation of the manuscript. All authors read and approved of the final manuscript.
